# Granal thylakoid structure and function: explaining an enduring mystery of higher plants

**DOI:** 10.1111/nph.18371

**Published:** 2022-08-03

**Authors:** Lianhong Gu, Bernard Grodzinski, Jimei Han, Telesphore Marie, Yong‐Jiang Zhang, Yang C. Song, Ying Sun

**Affiliations:** ^1^ Environmental Sciences Division and Climate Change Science Institute Oak Ridge National Laboratory Oak Ridge TN 37831 USA; ^2^ Department of Plant Agriculture University of Guelph Guelph ON N1G 2W1 Canada; ^3^ School of Integrative Plant Science Cornell University Ithaca NY 14853 USA; ^4^ School of Biology and Ecology University of Maine Orono ME 04469 USA; ^5^ Department of Hydrology and Atmospheric Sciences University of Arizona Tucson AZ 85721 USA

**Keywords:** cytochrome *b*_6_ *f* complex, electron transport, grana stacks, photosystems, stroma lamellae, thylakoid

## Abstract

In higher plants, photosystems II and I are found in grana stacks and unstacked stroma lamellae, respectively. To connect them, electron carriers negotiate tortuous multi‐media paths and are subject to macromolecular blocking. Why does evolution select an apparently unnecessary, inefficient bipartition? Here we systematically explain this perplexing phenomenon. We propose that grana stacks, acting like bellows in accordions, increase the degree of ultrastructural control on photosynthesis through thylakoid swelling/shrinking induced by osmotic water fluxes. This control coordinates with variations in stomatal conductance and the turgor of guard cells, which act like an accordion's air button. Thylakoid ultrastructural dynamics regulate macromolecular blocking/collision probability, direct diffusional pathlengths, division of function of Cytochrome  *b*_6_ *f* complex between linear and cyclic electron transport, luminal pH via osmotic water fluxes, and the separation of pH dynamics between granal and lamellar lumens in response to environmental variations. With the two functionally asymmetrical photosystems located distantly from each other, the ultrastructural control, nonphotochemical quenching, and carbon‐reaction feedbacks maximally cooperate to balance electron transport with gas exchange, provide homeostasis in fluctuating light environments, and protect photosystems in drought. Grana stacks represent a dry/high irradiance adaptation of photosynthetic machinery to improve fitness in challenging land environments. Our theory unifies many well‐known but seemingly unconnected phenomena of thylakoid structure and function in higher plants.

## Introduction

The chloroplast thylakoid is one of the most complex, highly organized biological membrane networks in nature. A striking feature of the thylakoid in higher plants is its bipartite architecture, which consists of appressed grana stacks and unstacked stroma lamellae. The grana stacks, which under electron microscopy look like columns of coins or accordion bellows, are helically intersected by unstacked stroma lamellae in right‐handed spirals with the lamellar surfaces slantly stretching away from the grana stacks (Austin & Staehelin, 2011; Bussi *et al*., 2019). Each granum contains a narrow, flat aqueous inner space (lumen), and neighboring grana are separated by a thin partition gap filled with stromal fluids. It has been suggested that across the partition gap, attractive van der Waals forces balance repulsive electrostatic and hydration forces to keep the grana together, although more research is still needed to resolve how these forces interact (Chow *et al*., 2005; Nevo *et al*., 2012; Puthiyaveetil *et al*., 2017). Each stroma lamella also encapsulates a luminal space. Typically, inside a chloroplast, several grana stacks are present and are connected by multiple sheets of stroma lamellae. The inner spaces of grana stacks and stroma lamellae are connected through slit apertures known as frets, located at the narrow margin of a granum, such that the whole thylakoid membrane network in a chloroplast encapsulates a single continuous aqueous lumen. Different stroma lamellae may be joined by left‐handed helices that further facilitate luminal communications across lamellae (Bussi *et al*., 2019).

Major protein complexes involved in light harvesting and electron/proton transfer have uneven distributions in thylakoid membranes (Anderson & Anderson, 1980; Danielsson *et al*., 2004; Nevo *et al*., 2012; Tikhonov, 2014; Koochak *et al*., 2019). Photosystem II (PSII) is mainly located in grana stacks. Photosystem I (PSI) and ATP synthase are mostly found in stroma lamellae. The cytochrome *b*_6_ *f* complex (Cyt) is evenly distributed between grana stacks and stroma lamellae. Photosystem I and ATP synthase favor stroma lamellae because both complexes have a headpiece (and also a stalk in the latter) that stick out of the stromal membrane surface and are too big to fit into the narrow partition gap between two overlapping grana (Nelson, 2009; Nevo *et al*., 2012). Photosystem II has a relatively flat surface exposure that fits into the partition gap of grana well, but it is not clear why PSII is excluded from stroma lamellae. The oxygen evolving complex (OEC) of PSII intrudes deeply into granal lumen (Kirchhoff *et al*., 2008). This intrusion is larger than the luminal intrusion of PSI or ATP synthase (Nevo *et al*., 2012). It is unclear whether the lamellar lumen is narrower than the vertical dimensions of the OEC and the granal lumen, thus discriminating against PSII. An alternative explanation is that the light‐harvesting complex II (LHCII) and the PSII–LHCII assembly may possess physicochemical properties that promote self‐association and the formation of grana stacks (i.e. grana stacks may simply form where the PSII–LHCII complexes are located). The cytochrome *b*_6_ *f* complex has a structure that fits comfortably in both grana stacks and stroma lamellae.

During the light reactions of photosynthesis, PSII and PSI trap excitation energy in parallel but must transport electrons in sequence to complete the linear electron transport (LET) from the ultimate donor (water) at the PSII end to the eventual acceptor (NADP+) at the PSI end. Both PSII and PSI are membrane‐bound and immobile. The electron transfer between them is achieved with the assistance of Cyt and two mobile electron carriers (plastoquinone (PQ) and plastocyanin (PC)). The cytochrome *b*_6_ *f* complex serves as a transit center between PSII and PSI, within which PQ and PC exchange electrons. Plastoquinone is hydrophobic, carries electrons on its surface, and diffuses within the lipid bilayer of the thylakoid between PSII and Cyt. Plastocyanin is soluble, protects its redox group within the molecular matrix, and diffuses in the aqueous lumen between the protrusion of Cyt at one end of its journey and that of PSI at the other end. If the Cyt located in grana stacks is used for LET, then the reduced PC must first diffuse in the granal lumen, then cross the slit apertures on grana margins to enter lamellar lumen, and finally deliver the electron to PSI. If the Cyt located in stroma lamellae is used for LET, then PQ would have to diffuse through the bent and therefore resistive membrane connections at frets to enter the lamellar membrane. The first scenario appears to be more likely as previous studies have found that PC may be the longer‐range carrier for LET as compared to PQ (Höhner *et al*., 2020).

The challenge for the diffusion of PQ and PC is that the thylakoid is a crowded place. The grana thylakoid membranes are heavily packed with PSII and light harvesting complexes, whereas the lumen is narrow relative to the size of PC and is subject to blockage by the OEC of PSII (Kirchhoff *et al*., 2017). It thus appears that neither PQ nor PC could diffuse freely in their respective domains, and there is a cost associated with the bipartite architecture of the thylakoid: a lower electron transport efficiency. This is puzzling because the bipartite system is apparently not even essential for oxygenic photosynthesis to occur. Cyanobacteria do not have such an architecture. Except for the recently evolved green algal group Charophyta, which have stacked grana broadly resembling those of higher plants, and *Chlamydomonas reinhardtii* chloroplasts, which have some loosely stacked thylakoid membranes (Anderson *et al*., 2008; Engel *et al*., 2015), grana stacks are absent in most algae.

Numerous hypotheses have been proposed to explain why evolution has selected the granum–lamella bipartite architecture for higher plants (Trissl & Wilhelm, 1993; Anderson & Aro, 1994; Chow *et al*., 2005; Mullineaux, 2005; Anderson *et al*., 2008; Pribil *et al*., 2014). Although no single hypothesis has stood out so far, a commonly accepted view postulates that grana stacking supports efficient packing of the light harvesting complexes of PSII (LHCII), maximal occupation of chloroplast space by the thylakoid membrane, and the prevention of excitation loss (spillover) from PSII to the deeper and faster energy trap PSI (Anderson & Anderson, 1980; Trissl & Wilhelm, 1993). This explanation works well for shade plants. Leaves strongly absorb blue and red light but scatter green and far‐red light. As a result, inside plant canopies, not only is the light intensity attenuated but also the light spectrum is altered. It is depleted in blue and red light but enriched in green and far‐red light compared to that in open environments (Hertel *et al*., 2011). Photosystem I is more sensitive to far‐red light than PSII, and the spectral distribution of light therefore favors PSI within plant canopies where shade plants live in natural environments (Anderson *et al*., 2008). Therefore, the survival and growth of shade plants can, in theory, be facilitated by efficient packing of LHCII, maximal occupation of chloroplast space by the thylakoid membrane, and prevention of excitation loss from PSII to PSI. However, it is not clear why shade plants, rather than sun plants, should dominate the evolution of photosynthetic apparatus when the light environment of the former is controlled by the latter. It has been suggested that different advantages of the bipartition may have been selected over time and collectively outweigh its deficiencies in photosynthesis, and it may be neither possible nor necessary to identify what is the evolutionally dominant force that drives grana formation (Nevo *et al*., 2012).

In this Viewpoint article, we propose a theory that unifies many well‐known but seemingly disparate phenomena of photosynthetic electron transport with key architectural characteristics of the chloroplast thylakoid in higher plants. Although the theory is conceptually rather simple, it can make numerous testable predictions. The foundation of our theory has two pillars – the ultrastructural flexibility of the bipartite thylakoid and the asymmetry of the photophysical and photochemical functions between PSII and PSI. These two phenomena have been reported previously, but their connection has not been widely recognized. We will first discuss them and then introduce the theory in which their connection is naturally established.

## Thylakoid ultrastructural flexibility and photosystem functional asymmetry in higher plants

The thylakoid in higher plants has a rather flexible volume. Electron microscopy has shown that thylakoids swell in the light and shrink in the dark (Kirchhoff *et al*., 2011; Kirchhoff, 2014). An almost doubling of lumen width from 5.0 to 9.4 nm has been observed in light, an expansion exceeding the size of PC (*c*. 4 nm, Li *et al*., 2020). Lumen swelling is coupled with thylakoid membrane stretching and narrowing of the partition gap between widened granal discs. This magnitude of light‐induced swelling has been predicted to significantly alleviate the restrictions of macromolecular crowding on the diffusion of free electron carriers (Kirchhoff *et al*., 2011).

The light‐induced dynamics of thylakoid ultrastructure are likely due to the osmotic water fluxes across the thylakoid membrane, from the stroma to the lumen, that are associated with lumen acidification and ion fluxes (Beebo *et al*., 2013; Li *et al*., 2020). The electron transport from PSII to PSI is coupled with a buildup of transmembrane electric gradient, with positivity on the lumen side due to the stroma‐to‐lumen proton translocation, charge separations at PSII and PSI, and the translocation of electrons from the lumen side to the stroma side within the Cyt complex. This electric gradient leads to ion movement via the ion channels in the thylakoid membrane towards the restoration of electroneutrality (Szabò & Spetea, 2017; Geilfus, 2018). The ion flux causes disequilibrium in water potential between the lumen and stroma, and therefore osmotic water flow across the thylakoid membrane, which in turn swells the lumen. In the dark, electron transport stops and the proton and electric gradients across the membrane dissipate, which leads to a reversal of osmotic water flow and a shrinking of the thylakoid. Osmotic swelling caused by differences in ion concentration is ubiquitous in both abiotic systems (e.g. clays, Madsen & Müller‐Vonmoos, 1989) and biotic systems (e.g. brains, Lang *et al*., 2014; various vesicles, Finkelstein *et al*., 1986).

The mechanism of light‐induced thylakoid ultrastructural dynamics is likely similar to that of the light‐induced buildup of turgor pressure in guard cells that opens the stomatal pores. The swelling and shrinking of guard cells are osmotically driven, with potassium as the primary osmolyte (Fischer, 1968). It is unclear whether the thylakoid has a primary osmolyte. If it does, a potential candidate is chloride, which is an essential nutrient to plants, although it is toxic at high concentrations (Geilfus, 2018). Chloride may be passively driven by the electrical gradient to the lumen through a voltage‐dependent chloride channel (Herdean *et al*., 2016; Li *et al*., 2021). However, the potential roles of cations cannot be excluded. In particular, potassium may enter the lumen via a potassium/proton antiport (Kunz *et al*., 2014), contributing to the luminal–stromal osmotic disequilibrium. Potassium is probably not the primary osmolyte for thylakoid swelling as this would cause loss of protons from the lumen and also direct or indirect competition with guard cells when both swell simultaneously. Just like mesophyll cells, guard cells contain chloroplasts. If potassium is the primary osmolyte for thylakoid swelling, the sequestration of potassium in the lumen may directly interfere with the uptake of potassium by vacuoles; vacuolar uptake of potassium and swelling are necessary for stomatal opening. Intermediary mesophyll cells and subsidiary cells act as ion reservoirs for guard cells. The sequestration of potassium in the lumen of the chloroplasts in the mesophyll cells may diminish its supply in these reservoirs and thus adversely affect the guard cells' capability to open and close stomata.

The structure of grana stacks facilitates the induction of thylakoid ultrastructural dynamics by osmotic water fluxes. The two flat surfaces of each thin granum are not connected to neighboring granal discs; rather, they are in direct contact with the stroma. Thus, grana stacks support high membrane densities, and yet all sides of each thin granum are in direct contact with the stroma, allowing rapid osmotic water exchanges between the lumen of each granum and the stroma. It is interesting to note that plastoglobuli are often observed to be attached to the granal margins. These plastid lipoprotein particles are formed by a membrane lipid monolayer that is directly connected to the outer leaflet of the granal disc membrane (van Wijk & Kessler, 2017). One can envisage that plastoglobuli may play the role of a lipid reservoir to support the thylakoid swelling and shrinking by feeding or taking up lipids as necessary in a carousel fashion (Kirchhoff, 2019), which would further enable the control of granal volumes via osmotic water fluxes across the thylakoid membranes.

Understanding the asymmetry of the photophysical and photochemical functions between PSII and PSI is fundamental to understanding the regulation of electron transport and photoprotection. Photosystem II and PSI are serially connected in the photosynthetic electron transport chain (ETC), with PSII in the head section and PSI in the tail section. They differ dramatically in at least five core functions that set apart their workloads and photoprotective priorities in volatile environments.

First, PSI is photochemically more efficient and has a bigger role for electron transport than PSII. For nonstressed plants, the maximum photochemical quantum yield of PSI is within the range of 0.94–0.98 (Hogewoning *et al*., 2012), while that of PSII is *c.* 0.83 across species (Björkman & Demmig, 1987; Johnson *et al*., 1993). This difference in photochemical efficiency is likely because PSI uses a bidirectional (symmetrical) mechanism for charge separation, whereas PSII employs a monodirectional (asymmetrical) mechanism (Caffarri *et al*., 2014). Both PSI and PSII contain a pair of primary electron acceptors – two special Chl*a* (*A*_o_) molecules in PSI and two pheophytin (Pheo) molecules in PSII – forming two branches of charge separation. In PSI, both branches are used with a high degree of efficiency; by contrast, in PSII, only one branch can separate electrical charge efficiently. As a result, PSI has faster overall trapping kinetics than PSII (Caffarri *et al*., 2014). If the absorbed energy is equally divided between PSI and PSII (Baker, 2008), then the electron transport rate via PSI must be higher than via PSII. Since every electron of the LET that passes through PSII must also go through PSI to support the Calvin–Benson cycle, the extra electrons that transfer through PSI mean that the alternative electron flows are stronger in PSI than in PSII. The alternative electron flows include cyclic electron transport (CET) around reaction centers and pseudo‐cyclic electron transport (PCET). Photosystem I CET cycles electrons back to the ETC through PQ along two different pathways that contribute to the acidification of the lumen and production of ATP but not NADPH. These pathways do not involve PSII but may compete with LET for access to Cyt and mobile electron carriers. The rate of PSI CET is not proportional to the rate of LET and varies with environmental conditions. It may range from undetectable under light limitation to as much as 40–70% of LET under light saturation (Laisk *et al*., 2010). In contrast to LET, which contributes to both ATP and NADPH, PSI CET contributes to ATP but not NADPH. Thus, PSI CET is crucial to generating proper ATP : NADPH ratios for the functioning of the photosynthetic apparatus in fluctuating environments and under stress (Alric & Johnson, 2017; Nawrocki *et al*., 2019). Photosystem I PCET uses electrons from PSI to reduce oxygen (the water–water cycle, also known as Mehler or Mehler‐like reactions), producing reactive oxygen species (ROS) and decreasing the excitation pressure of PSI. Photosystem I PCET can help protect PSI from photoinhibition if the produced ROS are quickly scavenged (Asada, 2006). A similar CET and PCET may exist in PSII, but this possibility has not been studied in depth. In typical LET, the reduced plastoquinone (plastoquinol, PQH_2_) is oxidized by Cyt. However, some PQH_2_ may cycle electrons back to the donor of PSII via a heme in the PSII complex, cytochrome *b*559, to form the PSII CET or be used to reduce oxygen via plastid terminal oxidase (PTOX) to form PSII PCET (Miyake & Okamura, 2003; Saroussi *et al*., 2019). Laisk *et al*.  (2015) suggested that the PSII CET and PCET may also involve proton translocation and help protect PSII from photoinhibition. Since the CET and PCET of PSI are better studied and likely more dominant, when we discuss CET or PCET here, we mean those of PSI unless otherwise stated.

Second, PSII is one of the most oxidizing enzymes in nature; by contrast, PSI is one of the most reducing (Caffarri *et al*., 2014). When its reduction by the OEC is impaired, the primary donor of PSII in the sustained oxidized state ($P_{680}^{+}$) can damage PSII reaction center proteins (Jegerschöld *et al*., 1990). By contrast, the oxidized primary donor of PSI ($P_{700}^{+}$) is a highly efficient nonphotochemical quencher, which protects PSI by dissipating excess excitation energy into harmless heat (Bukhov & Carpentier, 2003; Sonoike, 2011).

Third, whereas the allocation of excitation energy to PSII photochemistry is directly regulated by the proton concentration in the lumen via nonphotochemical quenching (NPQ; Ruban, 2016), a corresponding lumen acidity‐mediated regulation has not been found for PSI. Ballottari *et al*. (2014) reported that zeaxanthin efficiently quenched fluorescence in PSI particles extracted from an Arabidopsis mutant, implying that a PSII‐type NPQ process might also occur in PSI. However, Tian *et al*. (2017) demonstrated that in wild‐type Arabidopsis, no zeaxanthin‐dependent NPQ existed in PSI, suggesting that the finding of Ballottari *et al*. (2014) was specific to the mutant they studied.

Fourth, PSII and PSI differ in the cost and speed of repairing photodamage. Oxidative damage to PSII is mainly sustained by its D1 subunit, which turns over rapidly. The photodamaged D1 is replaced while the rest of the subunits of PSII are reused in the repair cycle (Kato *et al*., 2018). By contrast, oxidative damage to PSI degrades all its core subunits, none of which are re‐used in the repair, and all of which must be regenerated and reassembled (Caffarri *et al*., 2014). As a result, repairing photodamage to PSI is much more costly, less efficient, and much slower than repairing photodamage to PSII (Sonoike, 2011).

Fifth, the photodamaged PSII protects PSI; by contrast, photodamage to PSI increases the risk of photodamage to PSII. Oxidative damage to PSII decreases LET, which increases the fraction of the PSI population in $P_{700}^{+}$, an efficient excitation energy quencher and a protector of PSI. Under this scenario, the protected PSI can continue to conduct CET and pump protons from the stroma to the lumen, which not only supports ATP production but also maintains the NPQ needed to control excitation pressure on PSII. By contrast, if PSI is photodamaged, electrons will accumulate at the acceptor side of PSII due to the decreased capacity for linear electron transfer to NADP+. The accumulation of reduced acceptors of PSII increases the production of ROS and increases the degree of photodamage to PSII (Vass, 2012). Furthermore, photodamage to PSI results in decreased CET, which may weaken the transmembrane pH gradient, reduce NPQ, and increase excitation pressure on, and photodamage to, PSII.

The five functional asymmetries discussed here all suggest that it is physiologically and therefore evolutionarily advantageous to place the focus of photosynthetic safeguarding mechanisms on the functionally more valuable PSI, rather than on PSII. This indeed appears to be the strategy that higher plants have taken for photoprotection (Larosa *et al*., 2018; Alboresi *et al*., 2019).

## A unifying theory of thylakoid structure and function and its predictions

We theorize that the physiological advantages of the granum–lamella thylakoid are rooted in its ultrastructural flexibility, which ideally supports the PSII–PSI functional asymmetry and the coordination between electron transport and gas exchange, enabling the sessile higher plants to survive and grow in fluctuating, stressful land environments where water may be lacking for unpredictable periods of time. Specifically, we propose that photosystem segregation, functional asymmetry, and the environmentally inducible thylakoid ultrastructural dynamics via osmotic water fluxes together constitute a key, previously overlooked, multi‐function electron transport control/photoprotection mechanism. We call this mechanism the ultrastructural control to distinguish it from the much‐studied mechanisms of NPQ and photosynthetic control of PQH_2_ oxidation by Cyt (Foyer *et al*., 2012).

Grana stacks determine the capacity of the ultrastructural control. Just like an accordion uses bellows, airflow, and air pressure to increase the degree of control over the articulation of sound when playing melodies and accompaniments, the photosynthetic machinery uses grana stacks, osmotic water flux, and osmotic pressure to increase the degree of control on LET, CET, the ATP : NADPH ratio, and photoprotection. Because PSII and PSI are distantly separated from each other, with PSII in grana stacks and PSI in stroma lamellae, and the electron exchange centers (Cyt) are distributed evenly between them, the capacity of the ultrastructural control is maximized for a given architecture of the thylakoid in a chloroplast. The ultrastructural control interacts with and complements NPQ and photosynthetic control of Cyt activity to balance the light and carbon reactions. It has a built‐in homeostatic capability to cushion the impacts of fluctuating environments. If grana stacks are analogous to the bellows of an accordion, then guard cells are analogous to its air button. Because the thylakoid and guard cells both use osmotic mechanisms to control their volumes, their dynamics can be synchronized by their shared responses to environmental conditions, allowing the capacity for electron transport to be in balance with the capacity for gas exchange through the stomata. This is similar to how an accordionist synchronizes the control of the volume of the bellows and the openness of the air button to play tunes. Our theory suggests that the wide‐ranging ultrastructural control enabled by the granum–lamella architecture is the driving force behind the evolution of the bipartite thylakoid in higher plants, so that the descendants of algal progenitors were able to adapt to life on land, where irradiance can fluctuate between high and low in an instant and water availability is erratic. This idea is elaborated on in the sections that follow.

### Components of the ultrastructural control

The ultrastructural control consists of at least six regulatory components, each with associated impacts on LET, CET, the ATP : NADPH ratio, and photoprotection: macromolecular blocking and collision probabilities between electron carriers and protein complexes and between carriers themselves; the direct diffusional pathlengths of PQ, PC, and ferredoxin (Fd); regulation of pH values in both the granal and lamellar lumens via the osmotic water fluxes across the thylakoid membrane; division of Cyt function between LET and CET; separation of the dynamics of proton concentration in granal and lamellar lumen compartments; and coordinated thylakoid and guard cell ultrastructural dynamics.

#### Macromolecular blocking and collision probability

The integral protein complexes (e.g. PSII, PSI, Cyt, and ATP synthase) act as obstacles to the diffusion of mobile electron carriers. For example, Blackwell *et al*. (1994) found that the diffusion coefficient of PQ in thylakoid membranes is two orders of magnitude lower than that in artificial lipid vesicles free of proteins. The importance of macromolecular blocking for electron transport has been recognized in previous studies (Kirchhoff *et al*., 2011; Höhner *et al*., 2020). As the thylakoid swells as a result of osmotic water influx, macromolecular blocking and the collision probability between carriers and protein complexes and perhaps also between carriers themselves will decrease because the carriers have more room to move around freely in the thylakoid membrane (PQ) or in the lumen (PC), which facilitates the delivery of electrons from PSII to Cyt to PSI.

#### Direct diffusional pathlengths

Li *et al*. (2020) observed that under illumination, the granal and lamellar lumen widths and the repeat distance (the distance between the center planes of two neighboring grana or two neighboring stromal gaps) increased, whereas the thickness of the bilipid layer and the width of stromal gaps decreased. This suggests that granal and lamellar lumen volumes increase and the thylakoid membranes are stretched. Since the force of swelling, which is due to osmotic pressure against thylakoid membrane elasticity, is applied from the inside of every granum in all directions, the diameter of the grana should also increase. As discussed earlier, the osmotically‐induced thylakoid swelling can be coupled with feeding of lipids from the plastoglobuli attached to granal margins. Therefore, we can infer that a swollen thylakoid means that the nearest‐neighbor distances between PSII and Cyt, and between Cyt and PSI, increase. These nearest‐neighbor distances define the shortest diffusional pathlengths free of any collision. Thus, PQ within the core of the lipid bilayer and PC in the lumen will have to diffuse across greater distances for electron delivery, but will do so unhindered. The swelling of the thylakoid may also affect the diffusional pathlength of Fd, which is the mobile carrier in the stroma for both LET and CET. The exact point at which the cyclic electrons enter back into the ETC around PSI is still uncertain. The properties of Cyt are consistent with those of the long‐sought ferredoxin‐quinone reductase (FQR), and Cyt may very well be this elusive enzyme (Nawrocki *et al*., 2019; Malone *et al*., 2021). It is reasonable to assume that the re‐entry point of cyclic electrons is at Cyt, or at least within the proximity of Cyt, which as the thylakoid swells should become, on average, more distant from PSI.

#### Regulation of luminal proton concentrations by osmotic water fluxes

Conventionally, the dynamics of luminal proton concentration have been discussed exclusively in the context of the direct addition of protons into the lumen via water splitting by the OEC and pumping at Cyt or removal through the ATP synthase. This is a solute (H^+^)‐based perspective. However, a solvent (H_2_O)‐based perspective might also need to be considered if the extent to which the lumen volume can change is as large as has been reported recently (Li *et al*., 2020). The osmotic influx of water simultaneously dilutes the proton concentration in the lumen and concentrates protons in the stroma. The osmotic efflux of water from the lumen to stroma does exactly the opposite. The solute‐ and solvent‐based perspectives can occur simultaneously; their net effect controls the transmembrane proton concentration gradient and its far‐reaching impacts on photosynthesis.

#### Division of function of cytochrome *b*_6_ *f* complex between LET and CET

The even distribution of Cyt between grana stacks and stroma lamellae coupled with the separate confinement of PSII and PSI in these two domains has implications for the division of Cyt function between LET and CET. Because PQ would have to overcome the resistance at the bent membrane at the frets to access Cyt in stroma lamellae, LET should be structurally compelled to favor the use of Cyt in grana stacks by PQ (Joliot & Johnson, 2011). Likewise, CET should favor the use of Cyt in stroma lamellae by Fd due to its proximity to PSI. The increased direct diffusional pathlengths of electron carriers resulting from thylakoid expansion may further facilitate the exclusive use of Cyt in grana stacks by LET and Cyt in stroma lamellae by CET.

#### Separation of the dynamics of proton concentration in granal and lamellar lumen compartments

Although the thylakoid lumen is a continuous space, the granal lumen and the lamellar lumen are connected via the slit apertures at the frets located at narrow grana margins, effectively forming two relatively independent compartments. Because of the division in Cyt function between LET and CET discussed above, protons in the granal lumen compartment are primarily sourced from LET (and possibly also from the CET and PCET of PSII; Laisk *et al*., 2015). By contrast, the lamellar lumen compartment acquires protons primarily from diffusion from the granal to the lamellar lumen and also from the CET and PCET of PSI. This source difference partially decouples the dynamics of proton concentration in the granal lumen from that in the lamellar lumen. In the grana, the proton concentration dynamics are more closely coupled with the activity of PSII, whereas the dynamics of lamellar proton concentrations are more closely coupled with the activity of PSI. The movement of protons from the thylakoid lumen to the stroma is primarily channeled by ATP synthase, although exchanges via a K^+^/H^+^ antiport are possible (Kunz *et al*., 2014). Since the ATP synthase is mostly located in stroma lamellae, protons produced by LET would need to diffuse from the granal lumen into the lamellar lumen to join the protons from the CET of PSI to contribute to the synthesis of ATP. This necessarily means that there is a proton concentration gradient from the granal to the lamellar lumen, with higher concentrations in the granal lumen. This between‐lumen compartment proton gradient exists simultaneously to, and interacts with, the well‐known transmembrane proton gradient to affect ATP production. The partial decoupling of the proton concentrations between the granal and lamellar lumen compartments has important implications for the independent regulation of LET and CET, as discussed in the ‘Functions of the ultrastructural control’ section.

#### Coordinated thylakoid and guard cell dynamics

We are not aware of any simultaneous observations of the dynamics of thylakoid and guard cells, and can only infer their coordinated and perhaps even synchronized dynamics from the fact that both swell in light and shrink in darkness via osmotic mechanisms. The same environmental factors that affect guard cell turgor and stomatal conductance, including light, temperature, CO_2_ concentration, and water availability (Kollist *et al*., 2014; Buckley, 2019), should also affect the dynamics of thylakoid ultrastructure, given that both are controlled by the disequilibrium of water potential with their respective surroundings inside the leaf. The use of a cation as the primary osmolyte by guard cells and the likely use of anions as osmolytes by the thylakoid ensure that the osmotic processes in the chloroplast thylakoid of mesophyll cells and in the guard cells complement each other, and their coordinated swelling/shrinking do not compete for the same osmolytes in the apoplast of the leaf. This also helps maintain electroneutrality in the mesophyll.

### Functions of the ultrastructural control

The six components of the ultrastructural control discussed immediately above can serve a wide range of functions in photosynthesis in higher plants. The time constants of processes in the photosynthetic machinery range from picoseconds in the light harvesting and excitation transfer in the light reactions (Blankenship, 2014) to several tens of minutes in stomatal conductance (Vialet‐Chabrand *et al*., 2017) and the activation of Rubsico (McNevin *et al*., 2006) in the carbon reactions. Since NPQ and the photosynthetic control of Cyt activity depend on the operation of the rather sluggish carbon reactions, additional mechanisms are needed to protect the photosynthetic machinery from overreduction of the ETC and potential photooxidative damage caused by the extremely fast light reactions when the Calvin–Benson cycle is not ready during the dark to light transition. In algae and cyanobacteria, the photoreduction of oxygen (the Mehler reaction) can support a significant fraction of electron transport and plays the role of a safety valve for excessive electrons (Asada, 2006). However, higher plants, and angiosperms in particular, have much lower capacities for using oxygen as alternative electron acceptors (Badger *et al*., 2000; Shirao *et al*., 2013). Therefore, the thylakoid shrinking in the dark may play a necessary role in preemptively protecting PSI: when light levels increase again, the heavy macromolecular blocking and high collision probability between carrier and carrier, and between carrier and complex (e.g. LHCII, OEC), can slow the electron transfer from PSII to PSI before the Calvin–Benson cycle is fully activated and NPQ and photosynthetic control of Cyt activity can adequately regulate electron transport.

When water is not limiting, osmotic water fluxes can regulate macromolecular blocking, collision probability, and direct diffusional pathlengths to a level such that the reduced (negatively charged) PQ, PC, and Fd become effective reservoirs of electrons to cushion rapidly fluctuating light environments and yet can still deliver electrons at a rate consistent with the activation level of the Calvin–Benson cycle and the CO_2_ availability in the vicinity of Rubisco. For example, when more electrons are produced under high light than can be consumed by the carbon reactions, these extra electrons can be temporarily carried around by PQ, PC, and Fd; when fewer electrons are produced during low light than are needed by the carbon reactions, the electrons stored in PQ, PC, and Fd can make up the difference. For PQ, PC, and Fd to act as effective electron reservoirs, these carriers must be able to move around freely, which requires the ultrastructural control not to be limited by a lack of osmotic water fluxes across the thylakoid membrane. Conceivably, the ultrastructural control could even act like stanchion lines for queues at airports, to facilitate orderly electron delivery in fluctuating environments. Under drought when thylakoid swelling is limited by a lack of osmotic water fluxes, the increased macromolecular blocking and collision probability decrease electron delivery from PSII to PSI, which focuses photodamage on the former to protect the latter.

The regulation of luminal proton concentrations via osmotic water fluxes may serve as a mechanism to increase the electron transport rate and therefore the photosynthetic rate during periods of high water availability and yet protect photosystems during water stress. The luminal acidity controls the development of the dominant high energy component of NPQ (qE) (Murchie & Ruban, 2020) and the activity of Cyt for electron transport (Malone *et al*., 2021). When water is readily available, the osmotic water influx slows the buildup of luminal acidity. This has the effect of limiting the development of NPQ and relaxing the suppression of Cyt activity, thus facilitating LET. The easing of the suppression of Cyt activity also helps CET. Under water stress, however, the lack of osmotic water fluxes enables a faster buildup of luminal acidity and promotes the development of NPQ and the suppression of Cyt activity. The increased NPQ reduces the excitation pressure on PSII, which, together with the suppression of Cyt activity for LET, protects PSI in water‐stressed plants.

The division of Cyt function between LET and CET and the partial decoupling of the proton concentrations between the granal and lamellar lumen compartments enable differential regulation of LET and CET, and flexible adjustment of the ATP : NADPH ratio in response to variations in environmental conditions. Linear electron transport results in the production of both ATP and NADPH. Cyclic electron transport produces ATP but no NADPH and has a primary role in ensuring a balanced ATP : NADPH ratio for the Calvin–Benson cycle and other metabolisms and in providing additional protons to the lumen to regulate NPQ and Cyt activity under fluctuating environmental conditions (Joliot & Johnson, 2011). The requirements for regulation are not the same for LET and CET and are achieved via different mechanisms. The importance of CET increases with the demand for ATP relative to NADPH, which is stronger under conditions such as extreme temperatures, low CO_2_, or drought (Yamori & Shikanai, 2016). Linear electron transport results in a fixed ATP : NADPH ratio. However, the ATP and NADPH produced by the light reactions support many metabolic pathways, including carbon assimilation, photorespiration, the reduction of nitrogen and sulfur, and the synthesis of proteins and volatile organic compounds, etc. These different pathways, which vary with environmental conditions, require different ATP : NADPH ratios (Kramer & Evans, 2011). As a result, the relative proportions of LET and CET must vary in response to environmental variations in order to produce dynamic ATP : NADPH ratios that meet the overall requirements of stromal metabolism. For example, CO_2_ assimilation and photorespiration together require an ATP : NADPH ratio of (3 + 7Γ*/*C*_c_) : (2 + 4Γ*/*C*_c_), where Γ* is the CO_2_ compensation point and *C*_c_ is the CO_2_ partial pressure in the stroma. As temperature increases, which increases Γ*, or *C*_c_ decreases in response to stomatal closure in drought, this ratio will increase (Kramer & Evans, 2011). Therefore, decoupling the regulation of LET and CET by luminal pH, even if only partially, will facilitate the adjustment of the ATP : NADPH ratio.

Because of the division in Cyt function between LET and CET and the partial decoupling of the proton concentrations between the granal and lamellar lumen compartments, the levels of NPQ and the granal Cyt activity and therefore LET are primarily regulated by the proton concentration in the granal lumen compartment. Similarly, the lamellar Cyt activity and therefore CET are primarily regulated by the proton concentration in the lamellar lumen compartment. The cross‐compartment influence depends on the granal to lamellar proton concentration gradient. This separation avoids a coerced indiscriminate regulation of LET and CET in a common direction when environmental stresses require them to be regulated in different directions. Additionally, the granal to lamellar lumen pH gradient may facilitate the regulation of the ATP : NADPH ratio to cushion the effects of environmental fluctuations. For example, an ATP : NADPH ratio lower than is needed by the current environmental condition is likely associated with a stronger than desirable LET or a weaker than desirable CET; under this condition, a steep proton concentration gradient would be present between the granal and lamellar lumens to accelerate the diffusion of protons from the granal lumen compartment towards the lamellar lumen compartment, where the ATP synthase is located, therefore increasing ATP production. Conversely, an ATP : NADPH ratio higher than is needed is likely associated with a weaker than desirable LET or stronger than desirable CET; under this condition, a gentler granal to lamellar luminal proton concentration gradient would be present, which slows the diffusion of protons from the granal to the lamellar lumen compartments, therefore decreasing ATP synthesis. The granal to lamellar proton diffusion passively responds to the balance between the production of protons by the OEC and influx via Cyt in the grana, and the net rate of efflux via ATP synthase and influx via Cyt in the lamellae. These influxes and effluxes are in turn affected by the limiting statuses of ADP and inorganic phosphate for ATP synthesis and of NADP^+^ and H^+^ for NADPH production in the stroma. This results in a mechanism for the granal to lamellar proton diffusion to respond to the varying ATP : NADPH ratio in a way that is conducive to rebalancing this ratio. In this mechanism, an ATP : NADPH ratio that is too low indicates substrate (NADP^+^ and H^+^)‐limited NADPH production, whereas an ATP : NADPH ratio that is too high indicates substrate (ADP and Pi)‐limited ATP synthesis. In the former, the accumulation of protons in the granal lumen would result in a steep granal to lamellar proton gradient; by contrast, in the latter, the decrease in ATP synthesis would lead to an accumulation of protons in the lamellar lumen, weakening the proton gradient. In both cases, the change in the granal to lamellar proton gradient occurs in a direction that facilitates the rebalancing of the ATP : NADPH ratio with the demands of the Calvin–Benson cycle.

At the leaf level, the balance between the light and carbon reactions is essentially the balance between electron transport in the thylakoid and gas diffusion through stomatal pores. The coordinated or even synchronized ultrastructural dynamics between the thylakoid and guard cells complement NPQ and photosynthetic control of Cyt activity to ensure that the availability of electron transport products is matched with the supply of CO_2_ to Rubisco. This thylakoid–guard cell coordination may be even more crucial during drought. Ideally, as the guard cells lose their turgor to narrow stomata, which decreases the capacity for gas exchange and prevents excessive water loss from plants, the thylakoid should shrink to lower the capacity of electron transport and concentrate protons in the lumen via osmotic water efflux to upregulate NPQ and suppress Cyt activity, which protects the photosystems, particularly PSI.

### Explanation of observations and some testable theoretical predictions

For convenience, we call the theory presented here the ‘bellows’ theory, due to the analogy between the grana stacks in the photosynthetic machinery and the bellows in an accordion. The bellows theory can immediately explain the following observations:

(**1**) The grana–lamella bipartition is ubiquitous in land plants but rare in aquatic photosynthetic organisms (Anderson *et al*., 2012), primarily because water availability is more volatile and the need for ultrastructural control of electron transport and photosynthesis is stronger on land than in water. Under water stress, decreased osmotic water influx restricts the swelling of the grana stacks, which increases the macromolecular blocking and carrier collision probability, limits the dilution of luminal proton concentration by osmotic water influx, increases NPQ, and lowers Cyt activity. These changes all contribute to decreasing the LET to balance with the reduced gas exchange due to stomatal closure, which protects PSI. Also, without water shielding, irradiance is high for land plants but can fluctuate rapidly. The large electron cushioning capacity of grana stacks via osmotic water flux‐induced swelling and shrinking enables plants to cope with such light regimes. Thus, the bipartite architecture of the thylakoid reflects a dry adaptation of the photosynthetic machinery, with increased tolerance to fluctuating light intensity to improve the fitness of higher plants on land.
(**2**) Photosystem II is more frequently photodamaged than PSI (Larosa *et al*., 2018; Alboresi *et al*., 2019) because PSII is at the beginning of the ETC and cannot benefit directly from the photoprotection offered by the ultrastructural control of electron transport. By contrast, PSI is located at the tail section and benefits directly from the protection of this control.
(**3**) Shade leaves in the lower canopy have more grana per stack (i.e. taller grana stacks) than sun leaves in the upper canopy (Anderson *et al*., 2012). The light environments of both sun and shade leaves are affected by the seasonal cycle and clouds. However, the light environment of shade leaves is additionally affected by the often sudden (on the timescale of seconds) appearance and disappearance of sunflecks caused by upper plant canopies swaying in the wind (Way & Pearcy, 2012). Therefore, the light environment of shade leaves fluctuates more than that of sun leaves. Swelling/shrinking of the thylakoid by osmotic water flux alone, which presumably operates on the timescale of stomatal conductance (minutes), is not fast enough to buffer such fluctuations. Taller grana stacks in shade leaves provide larger volumes for electron carriers to cushion the rapid fluctuation of the light environment. This is much like using solar panels to generate electricity in places where there are frequent fogs or clouds, necessitating larger battery banks to smooth out the output of electric power. Additionally, the large volume of granal lumen in shade leaves increases the dilution of proton concentration by osmotic water influx, relaxing the limitation of NPQ and Cyt activity on LET, thus compensating for the effect of a light environment enriched in spectra favoring PSI.
(**4**) The capacity for diverting electron flow to oxygen is considerably smaller in higher plants than in algae and cyanobacteria (Badger *et al*., 2000; Asada, 2006; Shirao *et al*., 2013) because the ultrastructural control reduces the dependence of higher plants on the reduction of oxygen for photoprotection during dark to light transitions. Using oxygen as an electron acceptor is a risky photoprotection mechanism because it produces ROS which can stress cells oxidatively and must be scavenged quickly before damage occurs. Also, oxygen always competes with NADP+ for electrons, even when no photoprotection is needed. By contrast, the ultrastructural control produces no harmful byproduct and does not lead to electron loss. Therefore, ultrastructural control is a safer and more efficient photoprotection mechanism than the photoreduction of oxygen. It is likely that the invention of granal stacks may have paved the way for higher plants to evolve mechanisms to control electron leakage to oxygen.

In the spirit of Karl Popper's Falsification Principle, we use the bellows theory to make the following testable predictions, whose invalidation will falsify the whole theory:

(**1**) A redox reaction‐based photochemical model of electron transport cannot simultaneously predict the relationship between LET and the redox state of PSII under high and low light conditions if the light‐induced thylakoid swelling/shrinking is not explicitly represented.
(**2**) The light‐induced thylakoid swelling is positively correlated with the increase in stomatal conductance, allowing electron transport to be balanced with gas exchange through stomata.
(**3**) The ultrastructural control regulates electron transport before the thylakoid is fully swollen. After the thylakoid is fully swollen and therefore loses its ultrastructural control on electron transport, NPQ and photosynthetic control should dominate as the Calvin–Benson cycle is fully activated. This implies that the light‐induced thylakoid swelling is correlated with the development of NPQ under nonstress conditions during dark to light transitions.
(**4**) The light‐induced thylakoid swelling slows the development of NPQ due to the dilution of proton concentrations in the lumen by osmotic water influx. Nonphotochemical quenching develops more rapidly as the thylakoid approaches its full expansion and the osmotic water dilution of luminal proton concentration decreases.

## Topics for future studies

Among the photosynthetic eukaryotes, land plants are the most diverse, both morphologically and physiologically. Yet they all share the same overall grana–lamellae bipartite chloroplast thylakoid architecture, regardless of photosynthetic pathways, which is unique among oxygenic photosynthetic organisms. This architecture facilitates close coordination between chloroplast thylakoids and guard cells in their osmotically controlled ultrastructural dynamics to ensure that the energy supply (electron transport) meets the raw material supply (CO_2_ diffusion) for manufacturing sugar. The balance between electron transport and CO_2_ diffusion – the two most important processes of photosynthesis – is crucial for the safety and operation of the photosynthetic machinery in challenging land environments. The evolution of grana–lamellae thylakoid, in conjunction with that of stomata, may have played a fundamental role in the successful conquering by higher plants of regions that are dry and/or subject to high/fluctuating irradiance.

An immediate research priority is to test predictions 1–4 of the bellows theory. This effort will require the development of a mechanistic model to predict redox reactions and electron transport along the ETC. This model will need to explicitly consider the thylakoid ultrastructural dynamics induced by osmotic water fluxes in response to variations in environmental conditions, and the resultant impact on the diffusion of mobile electron carriers. Such a model can then be used in conjunction with joint measurements of pulse amplitude modulated (PAM) fluorometry and gas exchange to test predictions 1–4.

Other predictions may be formulated to continue to challenge the bellows theory. Points of interest include how drought, artificially induced decoupling of stomatal and thylakoid dynamics, or artificially induced destacking of grana might influence electron transport; the relative risks of PSII vs PSI photodamage; and photosynthesis under environmental conditions with contrasting patterns of fluctuation. Another key prediction of the bellows theory is that there is a significant pH gradient between the granal and lamellar lumens. This gradient may be directly measured with improved pH‐sensitive fluorescence probes in live cell organelles (Ma *et al*., 2017).

Previous studies (e.g. Li *et al*., 2020) only compared the thylakoid ultrastructural differences between dark and light conditions. Observations of the thylakoid ultrastructural dynamics in response to continuous variations in light intensity are needed to test and parameterize models of the thylakoid swelling/shrinking functions. Such observations should be made across species as the thylakoid ultrastructural dynamics may be a species‐specific process and may depend on the prevailing environmental conditions where the leaf resides. It would be interesting to investigate how these differences may be caused by architectural differences in the thylakoids of different species, for example, the height of the grana stacks and the number of grana stacks per thylakoid.

The bellows theory suggests that ultrastructural control is a safer and more efficient photoprotection mechanism than the photoreduction of oxygen, and the evolution of the grana stacks may be linked to the diminished electron leakage to oxygen in higher plants as compared to algae and cyanobacteria. However, there are phylogenetic differences within the higher plants with respect to their capacity to use oxygen as an alternative electron acceptor. Gymnosperms have a higher potential for photosynthetic electron flow to oxygen than angiosperms (Shirao *et al*., 2013). Also, the oxygen photoreduction mediated by flavodiiron proteins in cyanobacteria and algae is also present in gymnosperms but not in angiosperms (Ilík *et al*., 2017). Shirao *et al*. (2013) suggested that such differences may be due to the general habitat differences between gymnosperms and angiosperms, as the main group of the former (i.e. conifers) tends to occupy regions with more frequent cold temperatures. The speed of thylakoid swelling/shrinking may decrease due to the increased viscosity of water at low temperatures, which may weaken the efficiency of the thylakoid's ultrastructural control on electron transport and increase the dependency of gymnosperms on oxygen photoprotection. Another possibility is that there may be systematic differences in the thylakoid architecture between gymnosperms and angiosperms, which can lead to differences in the efficiency of ultrastructural control.

Plants increase the concentration of abscisic acid (ABA) in leaves during drought, which triggers the loss of osmolytes and a decrease in turgor of the guard cells, decreasing the stomatal conductance (Buckley, 2019). Can a drought‐induced increase in ABA concentrations also trigger osmolyte loss from the lumen and shrink the thylakoid? Answering these questions may lead to a better understanding of plant–water relations and differences in species drought responses.

Nonphotochemical quenching has been targeted for bioengineering to improve plant responses to shading induced by clouds and swaying canopies with the potential to increase photosynthetic efficiency and crop productivity (Kromdijk *et al*., 2016). Understanding how environmentally induced thylakoid ultrastructural dynamics affect the development and relaxation of NPQ will be important to the efficacy of this effort.

## Author contributions

LG planned, designed, and conducted the research and derived all equations. BG, JH, TM, Y‐JZ and YS conducted measurements. YCS checked the derivation of equations and contributed to numerical solutions and discussions. LG wrote the first draft and worked with the coauthors on the subsequent revisions of the manuscript. Note that the equations and measurements that inspired the development of the bellows theory are not included in this Viewpoint but will be presented elsewhere.
